# Potential drug targets in the kynurenine pathway to treat acute schizophrenia

**DOI:** 10.1192/bjo.2021.461

**Published:** 2021-06-18

**Authors:** Ayush Malhotra, Paul Manowitz

**Affiliations:** 1 Rutgers University Graduate School of Biomedical Sciences; 2 Rutgers Robert Wood Johnson Medical School

## Abstract

**Aims:**

Schizophrenia is a serious developmental psychiatric disorder with a neurodegenerative component that causes marked deterioration in social relationships and ability to work. Present treatments are not satisfactory. Meta-analysis of placebo-controlled studies in acute schizophrenia shows that only a minority of patients have a good response to current antipsychotic medications. Therefore, there is a need for more effective psychopharmacologic treatments for this disorder.

**Method:**

The purpose of this paper is to provide new interpretations of existing data to provide a scaffolding for the development of novel drug targets for the treatment of schizophrenia. The causes of schizophrenia are most likely heterogeneous and involve both genetic and environmental factors. The authors examined a wide range of purported causes of schizophrenia to identify a common biochemical pathway that would contribute to this disorder. This review specifically did not consider pathways that supported the dopamine hypothesis of schizophrenia since historically drugs focused on dopaminergic mechanism, as noted in the aims, have not been successful for many patients with schizophrenia.

**Result:**

Two prominent schizophrenia-associated factors that have been widely studied with significant supporting evidence are stress and inflammation. Stress and inflammation share a common biochemical pathway that converges on the kynurenine pathway of the metabolism of tryptophan, an essential amino acid. At one end of the pathway, recently hospitalized patients with schizophrenia have been found to have low plasma tryptophan levels, whereas chronic schizophrenics have not, suggesting stress- and/or inflammation-induced increased metabolism of tryptophan. At the other end of the pathway, there is increased level of cerebrospinal fluid kynurenic acid in patients with schizophrenia as compared with healthy controls. Salivary kynurenic acid is associated with stress intolerance in schizophrenia. Importantly, natural occurring compounds in this pathway have significant CNS effects that include neurotoxicity and altered neural transmitter behavior.

**Conclusion:**

Stress and inflammation, both associated as causes of schizophrenia, are linked by a common biochemical pathway involving kynurenine. Examination of specific elements of the kynurenine pathway may aid in the identification of drug targets for schizophrenia.

